# *Espeletia praesidentis*, a new species of Espeletiinae (Millerieae, Asteraceae) from northeastern Colombia

**DOI:** 10.3897/phytokeys.76.11220

**Published:** 2017-01-05

**Authors:** Mauricio Diazgranados, Luis Roberto Sánchez

**Affiliations:** 1 Natural Capital and Plant Health department, Royal Botanic Gardens, Kew, Wakehurst Place Ardingly, West Sussex, RH17 6TN, UK; 2 Departamento de Biología y Química. Universidad de Pamplona. Pamplona, Colombia

**Keywords:** Colombia, Compositae, Espeletiinae, Espeletia, frailejón, Millerieae, Norte de Santander, Santander, páramos, Presidente

## Abstract

A new species of *Espeletia* from the Páramo de Presidente in northeastern Colombia is described. The species is named *Espeletia praesidentis* after the name of the páramo, and it is dedicated to the President Juan Manuel Santos, for his persistent efforts in working for peace for Colombia. The new species is closely related to *Espeletia dugandii*, but differs in the shape and colour of the leaves and arrangements of the capitulescences. A large population was found, but its total extension is yet to be determine.

## Introduction

In 1932 the renowned Spanish botanist José Cuatrecasas visited for the first time a páramo in Colombia. From that moment he decided to study the frailejones (common name given to most of the *Espeletia* Mutis ex Bonpl. species), not knowing the taxonomic endeavor he was going to begin. Thirty-four years later he published the classification of the subtribe Espeletiiane Cuatrec. (Asteraceae: Millerieae) (Cuatrecasas 1976), and continued working on the group until his death in 1996. His masterpiece, the systematic study of the subtribe, represents his work for about 64 years, and was finally published in 2013, after almost two decades of further additions. The treatment, however, did not include the genus *Espeletiopsis* Cuatrec., because Cuatrecasas was not able to complete the work on it (Cuatrecasas 2013). The main reason for the delay was the difficulty of working on the group, defying species concepts and challenging collectors and curators to deepen their collecting techniques and studies.

In recent years, several new species have been published, and more new species are foreseen. At the moment the subtribe contains 8 genera, 144 species (including the new species described here), 17 subspecies, 22 varieties and 8 forms (Cuatrecasas 2013; Diazgranados 2012a; Diazgranados and Morillo 2013; Diazgranados and Sanchez 2013). It has been highlighted as one of the examples of the rapid radiations of the páramos (Madriñán et al. 2013), and phylogenetic approaches will bring further reorganizations within the subtribe (Diazgranados 2012b).

There are a number of reasons making the subtribe a difficult group for taxonomist. It is easily identified morphologically, with clear synapomorphies, unique to these plants. However, the variation between and within the species can be astonishing. Despite the notorious morphological variations, characters are often continuous rather than discrete, and species are identified by combinations of character states. This is evident, for example, in Cuatrecasas’ dichotomous keys, where paragraphs with various character states are needed to identify the species (Cuatrecasas 2013). There is frequent inter-specific and inter-generic hybridization with introgression. The presence of hybrids with three parental species has even been suggested (Cuatrecasas 2013; Diazgranados 2012a). Hybrid speciation can be also important, as genomic analyses are recently suggesting (Mavárez, J., unpubl. data). Genetic variability between species is very low, and genetic markers are not very useful for separating species (Diazgranados 2012b). Also, populations are very large, dominating the landscape of the páramos. This challenges unexperienced collectors, who collect rare individuals that can be hybrids or just mutants in the population, without reporting the population. Also, collecting frailejones appropriately is time-consuming and samples are bulky. Due to all of this, herbarium samples are often incomplete and can be misclassified and misidentified. Finally, because of the island effect of the páramos and the limited dispersal capability of frailejones, local (geographic) endemism is common (Diazgranados 2013). The previous reasons form together the ingredients for a perfect ‘taxonomic storm’, inviting review of the species concept for Espeletiinae. In this work we follow the *unified species concept* (De Queiroz 2007), applicable for rapid radiations in early states of divergence, where populations become phenetically distinguishable and diagnosable.

The genus *Espeletia*, as it is currently defined, contains 72 species (including the one described here), distributed from the páramos of Lara (Venezuela) to the páramos of Llanganates (Ecuador). Most of the species grow above the timberline, although some have been identified as pioneer species after disturbance and can grow in ecotones between the high-Andean forest and the páramos, in azonal páramos or in areas with secondary paramisation (Diazgranados 2013).

This genus is diagnosed by being caulirosulas normally monocaulous, with lateral dichasial capitulescences, with branches, leaves, and bracts opposite, at least in the proximal part; leaf bases open, flat; ray corollas yellow, cypselae epappose, and pollen grains with 11–18(–21) spines, (2–)4–7 µm long on equator. Within the genus Cuatrecasas (2013) proposed five sections (*Aristeguietana*, *Bonplandia*, *Badilloa*, *Espeletia* and *Weddellia*) and a dubious group (Aberrantes). However, molecular works have suggested the presence of two clades (one from Venezuela and one from Colombia-Ecuador), which includes other genera (Diazgranados 2012b).

## Methods

The Páramo de Presidente is located 28 km south from Chitagá (Norte de Santander), and it is considered part of the Páramos de Almorzadero-Santurbán complex. It can be reached on the road from Chitagá to Cerrito. For decades this area has been considered unsafe, and the flora of this páramo has not been studied well yet. Material of the new species was collected during an expedition of the authors in 2009, in which they met with left-wing armed members. Duplicates were distributed to COL, ANDES and HECASA. Additional duplicates will be distributed to other Colombian herbaria. Micrographs were taken by the first author at the Scanning Electron Microscopy Laboratory of the National Museum of Natural History, in Washington DC. Lauren Merchant from Saint Louis University provided the illustrations, which were funded by the Missouri Botanical Garden and the Smithsonian Institution.

## Taxonomy

### Espeletia praesidentis

Taxon classificationPlantaeAsteralesAsteraceae

Diazgr. & L.R.Sánchez sp. nov.

urn:lsid:ipni.org:names:77159581-1

**Figure 1.**
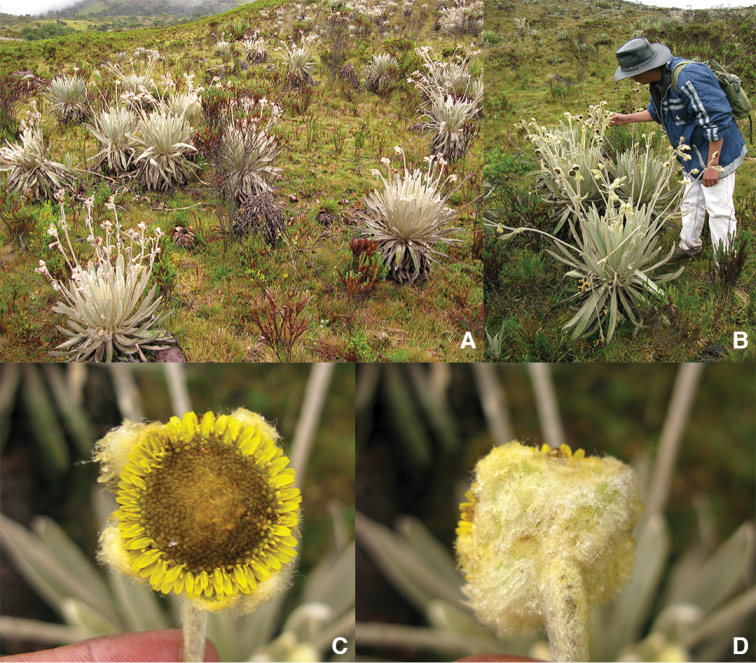
*Espeletia praesidentis*: **A** habitat, showing a large population **B** holotype collection (*M. Diazgranados* & *L.R. Sánchez 3865*), with stemmed rosette habit and very long capitulescences **C** frontal view of capitulum **D** dorsal view of capitulum.

**Figure 2.**
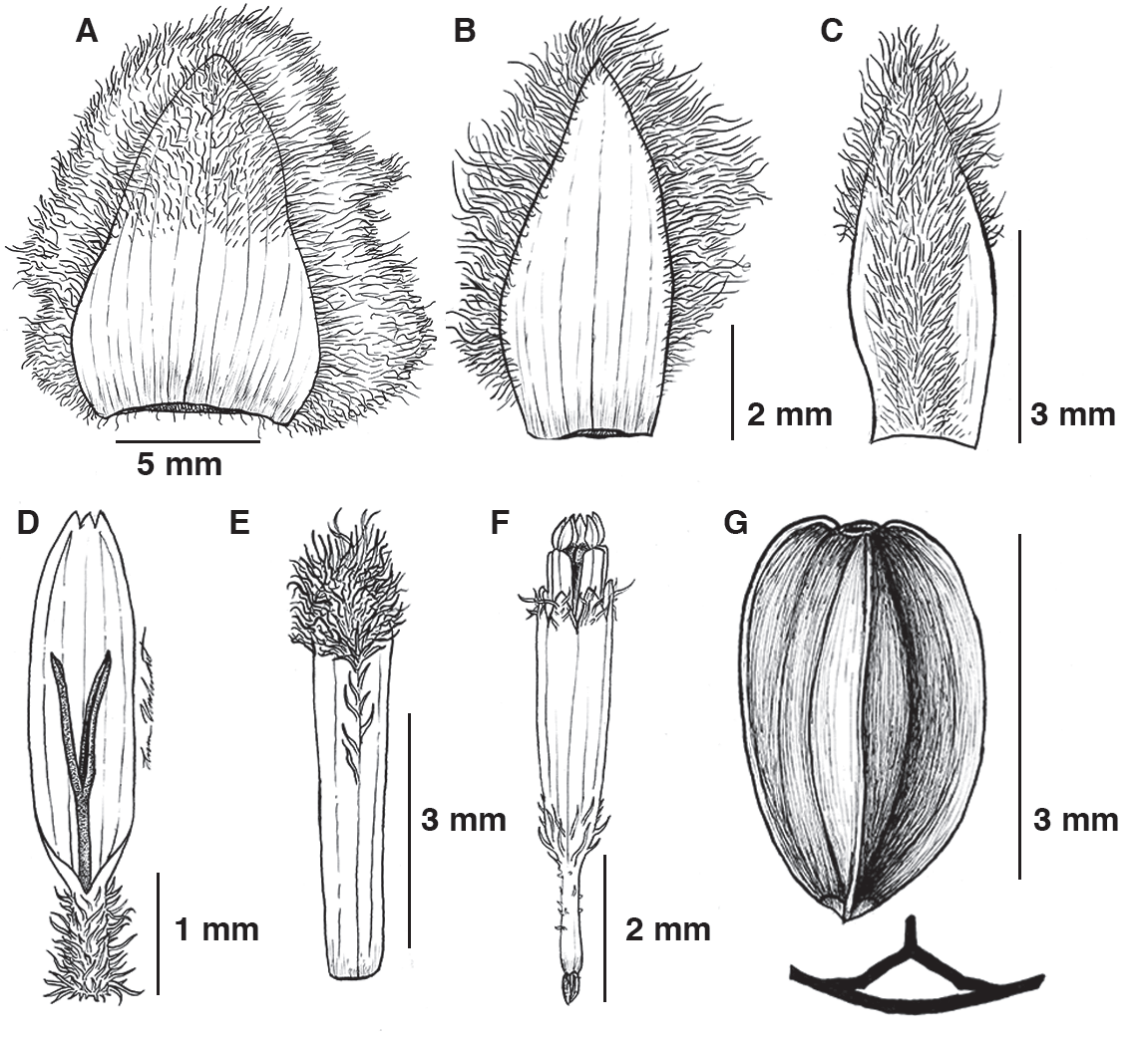
Illustrations of *Espeletia praesidentis*. **A** outer phyllary **B** Inner (sterile) phyllary **C** ray flower palea **D** ray flower **E** disc flower palea **F** disc flower **G** dorsal view of cypselae from ray flower. Illustrations made by Lauren Merchant.

**Figure 3.**
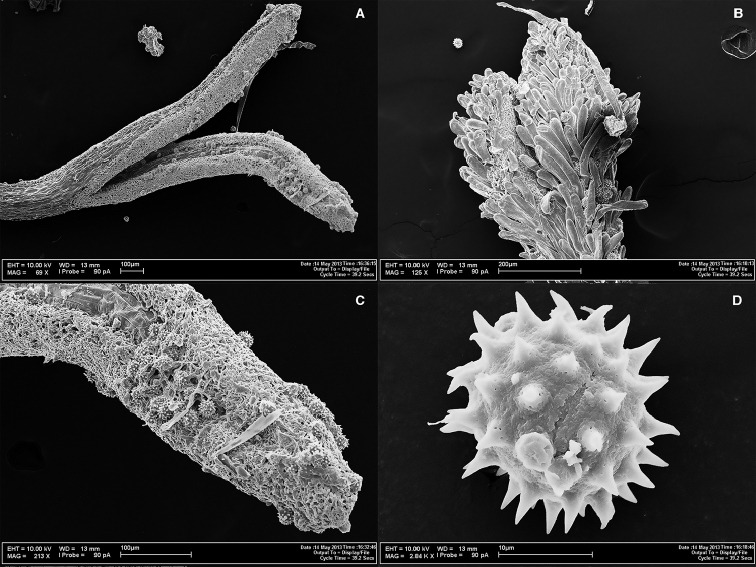
Photomicrographs of *Espeletia praesidentis*. **A** stigmatic branches of ray flower **B** stigmatic branches of disc flower with abundant papillae **C** detail of a stigmatic branch of ray flower with pollen grains **D** Pollen grain.

**Figure 4.**
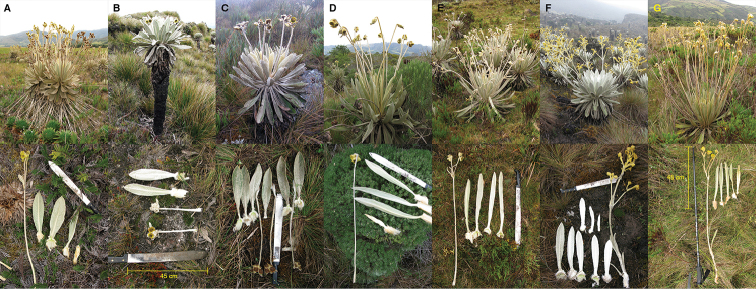
Comparison of similar *Espeletia* species. **A** *Espeletia brassicoidea* **B** *Espeletia canescens* **C** *Espeletia conglomerata* **D** *Espeletia dugandii* **E** *Espeletia praesidentis* **F** *Espeletia standleyana* **G** *Espeletia steyermarkii*. The hygrophilous and always monocephalous *Espeletia estanislana* was not included because of its very distinctive morphology. Above: plant habit; below: adult leaves with sheaths, and complete capitulescences.

**Figure 5.**
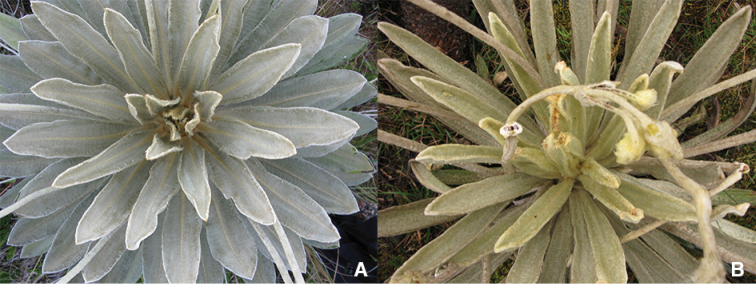
Comparison of rosette colours. **A** *Espeletia dugandii* **B** *Espeletia praesidentis*.

**Figure 6.**
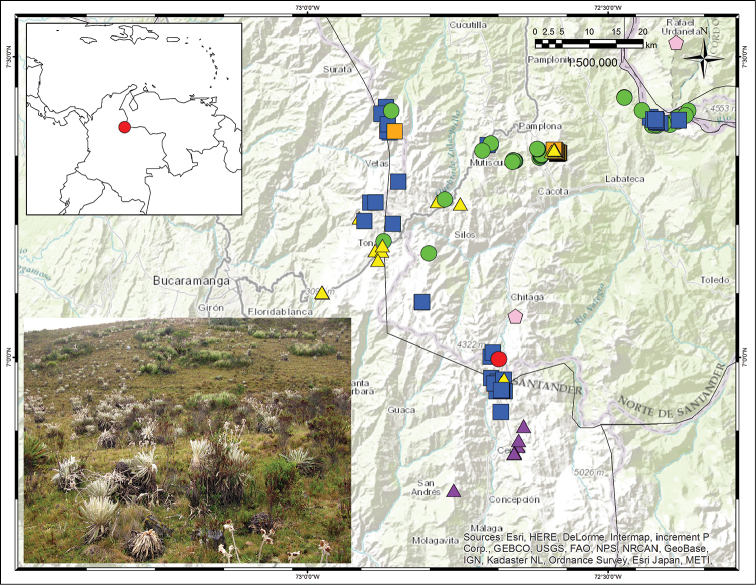
Distribution map showing the collection locality for *Espeletia praesidentis* (red circle), and collections of other Espeletia species found in the area: *Espeletia brassicoidea* (green circles), *Espeletia canescens* (orange squares), *Espeletia conglomerata* (blue squares), *Espeletia standleyana* (yellow triangles) and *Espeletia steyermarkii* (pink pentagons). Topographic map from Environmental Systems Research Institute (Esri), HERE, DeLorme, TomTom, Intermap, Increment P Corp., General Bathymetric Chart of the Oceans (GEBCO), United States Geological Survey (USGS), Food and Agriculture Organization (FAO), National Park Service (NPS), Natural Resources Canada (NRCAN), GeoBase, Institut Géographique National (IGN), Kadaster NL, Ordnance Survey, Esri Japan, Ministry of Economy, Trade and Industry of Japan (METI), Esri China (Hong Kong), Swisstopo, MapmyIndia, © OpenStreetMap contributors, and the GIS User Community.

Figures 1 , 2 , 3 , 4 , 5 , 6

#### Type.

COLOMBIA, Norte de Santander, Páramo de Presidente. En vía a Chitagá, llegando al páramo. En frailejonal-pajonal típico. Muy abundante. Caulirrósula. Alt. tot.: 0.8 m; alt. de la roseta: 0.4 m; inflorescencias: 2 maduras y 4 secas, con escapo desnudo, con 3–5 capítulos, cada uno de 2.1 cm de diámetro; hojas más angostas que otros individuos simpátricos. Alt. 3503 m, -72°40.8828'W, 6°59.8362'N. 3 Oct. 2009, *M. Diazgranados* & *L.R. Sánchez 3865* (holotype: COL; isotypes: HECASA and to be distributed).

#### Diagnosis.

Caulescent rosette of yellowish-whitish appearance, with leaf laminae linear or linear-obovate, naked scapes with long peduncles and 3(–5) capitula, small in diameter, disc paleae oblong, oblanceolate or narrowly obtrullate, very short yellow ray flowers, and lobes of disc corollas with hairs. Similar to *Espeletia dugandii*, but more yellowish, with much linear and narrower leaf laminae, much longer peduncles, smaller capitula and ray flowers, and disc corolla lobes with more hairs.

#### Description.

Caulescent polycarpic rosette of yellowish-whitish appearance (not cinereous), 0.8–1.5 m tall (including capitulescences), growing in grassland of páramo proper. Excluding reproductive parts, rosette 40–60 cm in diameter, on stems 0–40 cm tall (Fig. 1 A–B).

Leaves firm, coriaceous, rigid, erect; laminae linear or linear-obovate, apex acute to subacute (60–80°), base sessile, slightly pseudopetiole, attenuate, (38–)39–42(–45) cm × (3.0–)3.5–3.6(–4.2) cm, length to width ratio (10–)11–12(–15):1 (Figs 4 E, 5 B). Indumentum pale-yellowish in young leaves, becoming whitish in adult leaves. Adaxial face with indumentum whitish, lanose, costa pale-yellowish, visible, but secondary nerves invisible. Abaxial face with indumentum whitish, lanose, less abundant, costa more prominent, as well as secondary nerves, with deviation angles of 37–45°. Margins entire.

Leaf sheaths open, oblong to trapezoidal, coriaceous, 5–6 cm wide × 7–8 cm long; adaxially glabrescent, whitish, with 10–15 green anastomosing nerves; tawny abaxially, barbate, with hairs up to 15 mm long.

Capitulescences 5–15(–18) coetaneous, cymose, dichasial, axillary (lateral), erect, more than twice longer than the leaves, 100–120 cm long; indumentum abundant, villous, white. Scapes erect, firm, 80–100 cm long, 0.8–1.0 mm in diameter; naked, with no sterile bracts. Peduncles terete, 15–18 cm long, curving in the distal end, proximally with a free attachment. One pair of subtending bracts, linear, 8–10 cm long × 0.9–1.0 cm wide.

Capitula 3(–5) radiate, subglobose, nodding, 2.0–3.0 cm in diameter (including ray flowers) (Fig. 1 C–D). Involucre 2.0–2,5 cm wide × 1.0–1.6 cm high. Phyllaries in 2–3 series, ovate to ovate-triangular. Outer phyllaries surpassing the capitulum, 12–13 mm long × 8.0–9.0 mm wide (excluding hairs), apex obtuse to acute, adaxially glabrous with 10–20 visible nerves, abaxially villous, hairs 2–4.5 mm. Inner phyllaries 6.0–6.5 mm long × 2.8–3.3 mm wide, with indumentum villous white, hairs 1.0–2.0 mm long.

Ray flowers 80–90 in 2–3 series, yellow, ray corollas 3.5–4.5 mm long (excluding ovary). Ligules 3.0–3.5 mm long, elliptical or oblong, tridentate; tube hirsute, small, 0.2–0.4 mm in diameter and 0.5–1.0 mm long, yellow, the trichomes 0.2–0.3 mm long. Style 2.7–2.9 mm long × 0.14–0.2 mm in diameter, with stigmatic branches 1.0–1.5 mm long, without papillae in the distal portion. Cypselae oblong, triangular, 3.3–3.5 mm × 2.0–2.2 mm, glabrous, black. Ray paleae narrowly-ovate, 5.3–5.5 mm × long 2.0–2.1 mm wide, brownish, profusely villous.

Discs 1.5–2.6 cm in diameter. Disc paleae oblong, oblanceolate or narrowly obtrullate, 5.0–5.4 mm long × 0.8–1.1 mm wide, brownish, glabrous becoming villous in the distal portion. Disc flowers 300–400; corolla 5.0–5.2 mm long (excluding anthers and fruit); corolla throat 3.5–3.7 mm long, 1–1.1 mm wide when open, 5-lobed, lobes 0.45–0.55 mm long, with hairs; tube 1.5–2.0 mm long × 0.2–0.3 mm in diameter, glabrous, with a few hairs; anthers dark yellow, sometimes exceeding the corolla, slightly translucid, approximately 1–2 mm long and 0.3 mm wide. Pollen yellow when fresh, tricolporate, 17.5–19.5 μm in equatorial diameter (not counting spines); spines 70–74 total, 14–16 equatorial spines, 3.9–4.5 μm long, erect. Style 5.5–7.0 mm long × 0.14–0.17 mm in diameter, with stigmatic branches 0.6–0.7 mm long, broadening in the distal portion, 0.20–0.25 mm wide, papillose, papillae to 0.15–0.2(–0.4) mm long.

#### Distribution.

Endemic to Colombia. This species has been found only in the Páramo de Presidente (part of the great Páramo de Almorzadero), at elevations of 3400–3600 m (Fig. 6). The known area of distribution is about 2 km^2^.

#### Ecology.

A large population of several hundreds of individuals growing in the grasslands of the páramo proper was observed (Fig. 1, 6). Other *Espeletia* species found in the area are: *Espeletia brassicoidea* Cuatrec., *Espeletia canescens* A.C.Sm., *Espeletia conglomerata* A. C. Sm., *Espeletia dugandii* Cuatrec., *Espeletia standleyana* A. C. Sm., and *Espeletia steyermarkii* Cuatrec. (Fig. 6). *E praesidentis* can be found in slightly humid plains and on relatively drained slopes.

#### Etymology.

The specific epithet of this new species, “*praesidentis*”, taken from the locality where the species is found, is dedicated also to the President of Colombia, Juan Manuel Santos Calderón, for his persistent efforts to achieve peace with the guerillas FARC in Colombia, after 52 years of conflict. The Páramo de Presidente has been one of those places that has been closed to researchers for decades. With the peace agreement this and other places will be open for fruitful botanical explorations during the post-conflict times in Colombia. May this publication inspire the President to continue with further actions for the preservation of Colombian biodiversity.

#### Conservation status.

Despite seeing a relatively large population, this páramo area is not under any sort of protection, and there are signs of grazing activity. Also, very close there are extensive potato plantations in areas that were covered by páramo vegetation in the past. This combination of elements suggests that the species is probably *Critically Endangered* (CR, according to the IUCN criteria: extent of occurrence estimated to be less than 100 km^2^, habitat fragmentation, and likely decline of the extent of the páramo; http://jr.iucnredlist.org/documents/redlist_cats_crit_en.pdf), or *Critically Imperiled* (G1, according to NatureServe; http://www.natureserve.org/explorer/ranking.htm).

#### Discussion.

The páramos of Santander and Norte de Santander (Colombia) are considered one of the three centres of radiation for the Espeletiinae (Cuatrecasas 2013; Diazgranados 2012b). Probably because of the topographic complexity of these mountains and the longer time for evolution of these plants in this area with respect to other Colombian cordilleras, the overall diversity in the Santanderes is remarkable: 36 species belonging to 7 genera (all but *Carramboa*). New species continue to appear as collectors reach previously unexplored páramos, whilst our taxonomic understanding of the group improves.

In 1926–1927 two American botanists explored the vegetation of these mountains, Ellsworth Paine Killip (1890–1968) and Albert Charles Smith (1906–1999). Smith, who would became later the director of the National Museum of Natural History at the Smithsonian Institution, described years later 10 new species of *Espeletia* from those collections. Since then, no one has really visited the same places that these botanists explored, probably not even Cuatrecasas, who spent decades collecting the Espeletiinae in the páramos. Collectors of Espeletiinae know well that if they miss the slope or the mountain, they can totally miss the species they are looking for, because of the extreme local endemism of the group. As a consequence, Cuatrecasas (2013) made clear in his monographic work that the status of several taxa could be subject to changes with further collections.

*Espeletia praesidentis* exemplifies the lack of collections throughout the páramos of the region, and the challenges taxonomists have to face when studying this group. Cuatrecasas’s collections were often limited to the accessibility of roads in those years (1940–1980), and he never found the topolocality where Killip and Smith collected species such as *Espeletia conglomerata* and *Espeletia canescens*. In the remarks for *Espeletia canescens* of his treatment he said “Sometimes I have been inclined to consider *Espeletia canescens* as a local, extreme variation of *Espeletia conglomerata*. However, the scanty, authentic material of *Espeletia canescens* shows features that can justify its specific status […] On my 1973 trip, I did not have the time to walk from La Baja all the way to the highest spots at the opposite north end of the Páramo del Romeral, where Killip probably collected the type specimens of *Espeletia canescens*. Additional collections from the extreme section of the Páramo del Romeral may clarify the taxonomic status of *Espeletia canescens*” (Cuatrecasas, 2013, pag. 319). In the remarks for *Espeletia conglomerata* he said “*Espeletia conglomerata* as well as *Espeletia canescens* were described with type specimens from Páramo del Romeral between “3800 and 4200” m of altitude. However, according to recent maps, this páramo generally does not exceed 3800 m […]. My own collections represent several minor variations, as well as the typical form” (Cuatrecasas, 2013, pag. 316). In that moment Cuatrecasas was 70-year old, and clearly did not have time or possibilities to explored close areas were in recent years various new species have been discovered (e.g. *Espeletiopsis sanchezii* S. Díaz & S. Obando or *Espeletia diazii* Diazgr. & L. R. Sánchez). With no other material than his own collections, he first described morphological variations of his specimens as varieties (*Espeletia conglomerata* var. *macroclada* Cuatrec. and *Espeletia conglomerata* var. *pedunculata* Cuatrec.). Later, he decided to change the status to hybrids, both within *Espeletia conglomerata* × *Espeletia brassicoidea*, and synonymised *Espeletia brassicoidea* f. *contracta* Cuatrec. with *Espeletia conglomerata*. Also, he never published *Espeletia conglomerata* var. *lanceolata* Cuatrec. [*Nom. nud.*, *Carriker 34*].

*Espeletia praesidentis* differs notably from the type of *Espeletia conglomerata* (*Killip E. P. and Smith A. C. 18635*, see key below), and from the hybrids described from Cuatrecasas (2013). We believe *Espeletia praesidentis* cannot be considered a local variation or hybrid of similar or neighboring species for two reasons: 1) there is a large population of several hundreds of individuals; and 2) there are remarkable morphological differences between *Espeletia praesidentis* and the type collections of other species (as seen in Fig. 4). In this work we do not intend to propose a new categorization for hybrids and/or varieties of *Espeletia conglomerata*, and we recognize that hybrids can be easily spotted when sympatric species occur, but this clearly was not the case.

### Key to *Espeletia praesidentis* and other *Espeletia* species found in an area (*Espeletia brassicoidea*, *Espeletia canescens*, *Espeletia conglomerata*, *Espeletia dugandii*, *Espeletia standleyana* and *Espeletia steyermarkii*)

The most complete key for the genus has been published by Cuatrecasas (2013) and updated later in various publications (Díaz-Piedrahita and Pedraza 2001; Díaz-Piedrahita and Rodriguez-Cabeza 2008; 2010; Díaz-Piedrahita et al. 2006). This key is a simplification of the updated version of Cuatrecasas’ key, for the species of *Espeletia* found in an area of 50 km of radius.

**Table:** 

1	Proximal vegetative parts of capitulescences naked, completely lacking sterile leaves, occasionally above proximal parts bearing a few leafy bracts originating from the fertile parts and becoming sterile	**4**
1’	Proximal vegetative parts of capitulescences each with from several to 1 pair of sterile opposite leaves	**2**
2	Leaf laminae somewhat narrowed towards bases, but not obviously pseudopetiolate. Capitulescences with spreading, stout, thick branches and peduncles	***Espeletia standleyana***
2’	Leaf laminae narrowed toward bases into conspicuous pseudopetioles	**3**
3	Capitulescences monocephalous; capitula 35–70 mm in diameter	***Espeletia estanislana***
3’	Capitulescences paniculate, proximal fertile internodes and branches very long (18–44 cm), rather thin, erect, and fastigiated; with numerous capitula (>20), (16–)20–35 mm in diameter	***Espeletia steyermarkii***
4	Leaf laminae 7–14 cm wide, length to width ratio 1.3–5(–6):1, elliptic, obovate, oblong-obovate or oblong-elliptic, abruptly or gradually narrowed at bases; general appearance white. Capitulescences about 2× longer than leaves	***Espeletia brassicoidea***
4’	Leaf laminae narrow, 3.2–6 cm wide, length to width ratio (5–)6–11(–15):1, oblong-spathulate or oblong-elliptic, occasionally obtrullate, gradually attenuate to bases. Capitulescences longer than 2× the leaves	**5**
5	Leaf laminae (26–)34–40 cm × 4.3–5.5(–6.5) cm; ray corollas 14–18 mm long; tubes 4.5-6 mm long; capitula 3–4 cm in diameter	***Espeletia dugandii***
5’	Leaf laminae shorter or narrower; ray corollas and tubes much shorter; capitula smaller, 2–3.2 cm in diameter	**6**
6	Plant appearance yellowish; leaf laminae linear or linear-obovate, (38–)39–42(–45) cm × (3.0–)3.5–3.6(–4.2) cm, length to width ratio (10–)11–12(–15):1. Peduncles 15–18 cm long. Ray corollas 3.5–4.5 mm long; tubes 0.5–1.0 mm long	***Espeletia praesidentis***
6’	Plant appearance whitish or cinereous; leaf laminae oblong, spathulate or narrowly obovate-oblong, much shorter and wider. Much shorter peduncles (1–6 cm). Ray corollas 7–11(13) mm long; tubes 1.5–2.5 mm long	**7**
7	Leaf laminae more thinly coriaceous, flexible, obtuse or subacute at apex, 22–32(–38) cm × 3.2–5.5 cm; capitula usually glomerate on curled, contracted peduncles (rarely peduncles almost straight); disc corollas hairy and glanduliferous at the middle, at least some of the lobes with a few hairs.	***Espeletia conglomerata***
7’	Leaf laminae rather thickly coriaceous, obtrullate-oblong, angulate and acute at apex, 30–32 cm × 5.3–6.3 cm; capitula drooping or nodding on straight rigid peduncles and pedicels; disc corollas glabrous or subglabrous; lobes only with sparse glands.	***Espeletia canescens***

## Supplementary Material

XML Treatment for Espeletia praesidentis
